# The Relationship between Adiposity and Insulin Sensitivity in African Women Living with the Polycystic Ovarian Syndrome: A Clamp Study

**DOI:** 10.1155/2016/9201701

**Published:** 2016-09-08

**Authors:** Emmanuella Doh, Armand Mbanya, Jean Dupont Kemfang-Ngowa, Sama Dohbit, Mycilline Tchana-Sinou, Pascal Foumane, Olivier Trésor Donfack, Anderson S. Doh, Jean Claude Mbanya, Eugene Sobngwi

**Affiliations:** ^1^National Obesity Center, Yaoundé Central Hospital, Yaoundé, Cameroon; ^2^Faculty of Medicine and Biomedical Sciences, University of Yaoundé 1, Yaoundé, Cameroon; ^3^Yaoundé General Hospital, Yaoundé, Cameroon; ^4^Yaoundé Gynaecology, Obstetrics and Pediatrics Hospital, Yaoundé, Cameroon; ^5^Laboratory of Molecular Medicine and Metabolism, Biotechnology Center, University of Yaoundé 1, Yaoundé, Cameroon

## Abstract

*Objectives*. We aimed to assess the variation of insulin sensitivity in relation to obesity in women living with PCOS in a sub-Sahara African setting.* Methods*. We studied body composition, insulin sensitivity, and resting energy expenditure in 14 PCOS patients (6 obese and 8 nonobese) compared to 10 matched nonobese non-PCOS subjects. Insulin sensitivity was assessed using the gold standard 80 mU/m^2^/min euglycemic-hyperinsulinemic clamp and resting energy expenditure was measured by indirect calorimetry.* Results*. Insulin sensitivity adjusted to lean mass was lowest in obese PCOS subjects and highest in healthy subjects (11.2 [10.1–12.4] versus 12.9 [12.1–13.8] versus 16.6 [13.8–17.9], *p* = 0.012); there was a tendency for resting energy expenditure adjusted for total body mass to decrease across the groups highest in obese PCOS subjects (1411 [1368–1613] versus 1274 [1174–1355] versus 1239 [1195–1454], *p* = 0.306).* Conclusion*. In this sub-Saharan population, insulin resistance is associated with PCOS per se but is further aggravated by obesity. Obesity did not seem to be explained by low resting energy expenditure suggesting that dietary intake may be a determinant of the obesity in this context.

## 1. Introduction

Polycystic ovarian syndrome (PCOS) is one of the most common endocrine disorders among women of the reproductive age. It is commonly undiagnosed due to the fact that its symptoms are heterogeneous and seem to have no relation with each other [1].

Obesity is a common symptom of polycystic ovarian syndrome although its cause still remains unknown [2]. In some recent studies, evidence has brought forth a relationship between some types of obesity and an alteration in insulin sensitivity [3]. Android obesity is the most indexed in this alteration and this is the main type present in women living with PCOS [4, 5].

This alteration in insulin sensitivity is also an associated and common factor in the polycystic ovarian syndrome. It eventually leads to various comorbidities over time particularly hyperglycemia [6]. Several mechanisms though not well understood have been incriminated in the insulin resistance associated with PCOS with obesity as the leading cause. Also a decrease in insulin receptor autophosphorylation and tyrosine phosphorylation of insulin receptor substrate-2 induces impairment of the insulin signal pathway hence increasing insulin resistance [7, 8].

Central obesity is thought to be strongly involved in insulin resistance in these women but is the obesity the cause of this insulin resistance? If no, the insulin resistance may be independent of the obesity and directly related to the pathogenesis of PCOS which will require further investigation. This knowledge may reveal novel therapeutic targets for the management of this pathology which has a devastating effect on a woman's self-esteem due to its aesthetic, reproductive, and cultural manifestations in our African setting.

Several studies have been carried out worldwide in order to better understand insulin resistance in PCOS subjects using gold standard methods such as the euglycemic-hyperinsulinemic clamp. To the best of our knowledge no such study has been published in sub-Saharan Africa. As a result, we carried out this study to investigate the relationship between obesity and insulin sensitivity in Cameroonian women living with PCOS. We hypothesized that obesity and PCOS both independently and synergistically cause insulin resistance in Cameroonian women living with PCOS.

## 2. Research Design and Methods

### 2.1. Research Participants

We carried out a cross-sectional, comparative study at the Endocrinology and Metabolic Disease Unit of the Yaoundé Central Hospital, Yaoundé (Cameroon). We enrolled 24 women of reproductive age (18–45 years) among which were obese PCOS patients (*n* = 6), nonobese PCOS patients (*n* = 8), and healthy adults (*n* = 10).

Diagnosis of PCOS was confirmed according to clinical presentation, presence of multiple cysts in the ovaries during ultrasonography, and serum hormonal imbalance (elevated levels of testosterone and LH; low progesterone and estrogen levels; and normal FSH levels) in accordance with the Rotterdam criteria [9]. Women with amenorrhea (cycle interval ≥ 6 months) and/or midluteal progesterone level ≤15 nmol/L were considered anovulatory. Normal range was 2.5–10.2 U/L for FSH and 1.9–12.5 U/L for LH. Hyperandrogenemia was considered if significant clinical hirsutism was present or serum testosterone level ≥3.5 nmol/L or free testosterone ≥62 pmol/L.

Hirsutism was assessed by the modified Ferriman-Gallwey score, the gold standard for clinical evaluation of hirsutism, with a cut-off level of ≥8 [10].

Obesity was determined based on the WHO classification of adult underweight, overweight, and obesity according to BMI [11].

Pregnancy, breast feeding, intercurrent illness, the use of any medication that could affect insulin metabolism, smoking, recent infection less than ten days prior to inclusion, and creatinine clearance ≤60 mL/min/1.73 m^2^ were exclusion criteria. The study protocol was approved by the Institutional Research Ethical Committee of the Faculty of Medicine and Biomedical Sciences, Yaoundé, and by the institutional review board of the Yaoundé Central Hospital. All participants gave their informed consent in accordance with the Declaration of Helsinki.

### 2.2. Procedure

Clinical and anthropometric data were collected using a predesigned questionnaire before body composition analysis and functional tests. Insulin sensitivity was assessed using a 2-hour euglycemic-hyperinsulinemic clamp at 80 mU/m^2^/min. Resting energy expenditure was measured using indirect calorimetry.

#### 2.2.1. Body Composition Analysis

This was evaluated using bioelectrical impedance. It consisted of using an impedance meter (TANITA®, TANITA Corporation, 1-14-2 Maeno-cho, Tabashi-ku, Tokyo, Japan). This noninvasive test simply involves the placement of two electrodes under the person's feet and two electrodes in their hands. A low level, imperceptible electrical current is sent through the body. The device measures how this signal is impeded through different types of tissue. The weight is recorded automatically. The output variables include the percent of body fat, fat mass, fat-free mass, and bone mass. The coefficient of variation of the bioelectrical impedance is 3-4%.

#### 2.2.2. Indirect Calorimetry

The Korr® ReeVue indirect calorimetry (Korr Medical Technologies, Inc., Salt Lake City, UT 84120, USA) was performed after a fast of at least 3 hrs. Participants were required not to smoke, drink alcoholic beverages, or do sports 24 hrs prior to the exploration. They were installed in a supine position and rested 20 mins in the said position. The calibrated calorimeter then recorded their breathing over 10 minutes. The results were then printed expressing resting energy expenditure adjusted for total mass.

#### 2.2.3. Euglycemic-Hyperinsulinemic Clamp

After an overnight fast, participants were admitted into the Clinical Research Facility of the Endocrine Unit of Yaoundé Central Hospital. Participants were required not to do any sports 1 week prior to procedure. Rapid insulin (Actrapid® HM Novo Nordisk A/S, 2880 Bagsvaerd, Denmark) concentrated at 100 mU/mL installed in a syringe pump (Alaris® Medical Systems UK Ltd., Basingstoke RG22 4BS, UK) and 10% dextrose solution were infused via the right antecubital vein. Blood was sampled through the left antecubital vein. A priming dose of insulin was given over the first 10 minutes followed by a constant infusion rate of 80 mU/m^2^/min up to the 120th minute [12, 13]. The 10% dextrose solution was infused as from the 11th minute at variable rates modifiable every 5 minutes using an infusion pump (IVAC Corporation, Model 598, San Diego, California) with the aim of maintaining capillary blood sugar levels at 5.5 ± 0.5 mmol/L. Capillary blood sugar measurements were done with a glucometer and strips (OneTouch® Ultra® 2, LifeScan Europe, Division of Cilag GmbH International, 6300 Zug, Switzerland). Blood samples were collected at baseline and the 100th, 110th, and 120th min.

### 2.3. Calculations and Definitions

Insulin sensitivity was calculated when the coefficient of variations of glycaemia and insulinemia and the rate of glucose infusion were less than 5% by the *M* value (mg/min/kg) which represents glucose disposal rate during insulin infusion. *M* value was calculated as the rate of glucose infusion minus the space of correction (SC) of glucose and then was adjusted for lean body mass. During the last 20 minutes of the euglycemic-hyperinsulinemic clamp, glycaemia is not constant, and SC has been defined to adjust for variations in glucose infusion rate and glycemic levels. SC was calculated using difference of glucose levels at the beginning and the end of the steady state period multiplied by 0.095.

### 2.4. Statistical Analysis

Raw data from the questionnaires were entered and coded in EpiData version 3.1. The entered data was then extracted to STATA 12.0 (StataCorp, College Station, TX, USA) for analysis. Qualitative variables were presented as a count and percentage while continuous variables as median [interquartile range]. Medians were compared between groups by the Kruskal-Wallis and the Mann Whitney-Wilcoxon rank sum test. The threshold for significance was set at 0.05.

## 3. Results

The median [interquartile range] age of the individual groups of our study was 26 [23–30] years for obese PCOS patients, 27 [24–29] years for nonobese PCOS patients, and 23 [23-24] years for healthy subjects.

Table 1 shows the baseline clinical and biological characteristics of our study population. BMI was significantly higher in obese PCOS subjects than in the nonobese subjects who were higher than the heathy subjects (*p* = 0.0003). Also, the median fat mass (*p* = 0.0015) and lean mass (*p* = 0.0378) were significantly higher in obese PCOS subjects than in nonobese PCOS subjects which were equally higher than in healthy subjects. Other parameters significantly higher in obese PCOS subjects were waist circumference and body fat percentage. Our study populations had similar fasting plasma glucose and systolic and diastolic blood pressure.

Table 2 shows the feeding and physical activity patterns in our study population. A greater percentage of obese PCOS subjects eat fewer vegetables and more fruits than nonobese PCOS subjects and controls. They are also more involved in sporting activities.

Following the euglycemic-hyperinsulinemic clamp, the clamp-derived *M* value adjusted for lean body mass expressed in mg/kg/min was lower in nonobese PCOS subjects when compared to healthy subjects and even lower in obese PCOS subjects; *p* = 0.012 (Table 3).

**Figure 1:**
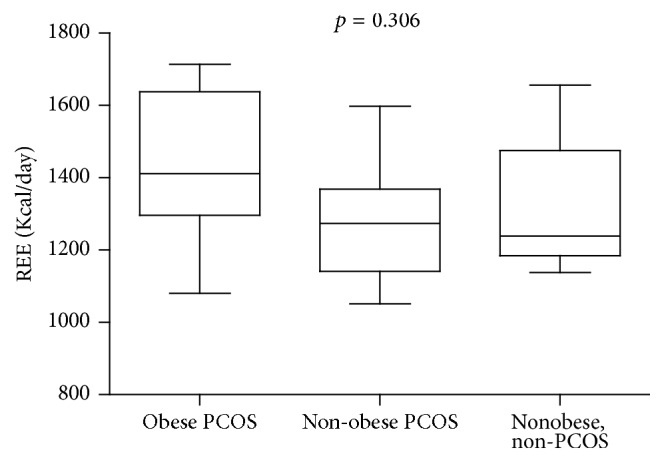
Resting energy expenditure adjusted for total body mass in Kcal/day across subgroups.

Indirect calorimetry measurements of resting energy expenditure were highest in obese PCOS subjects followed by nonobese PCOS subjects and lowest in healthy subjects but were not significant; *p* = 0.306 (Figure 1).

Table 4 shows the correlation between clinical parameters, biological characteristics, and the *M* value adjusted for lean body mass expressed in mg/kg/min in our study population. Among all the parameters, body mass index was inversely correlated with lean body mass adjusted *M* value (*r* = −0.56; *p* = 0.005).

**Figure 2:**
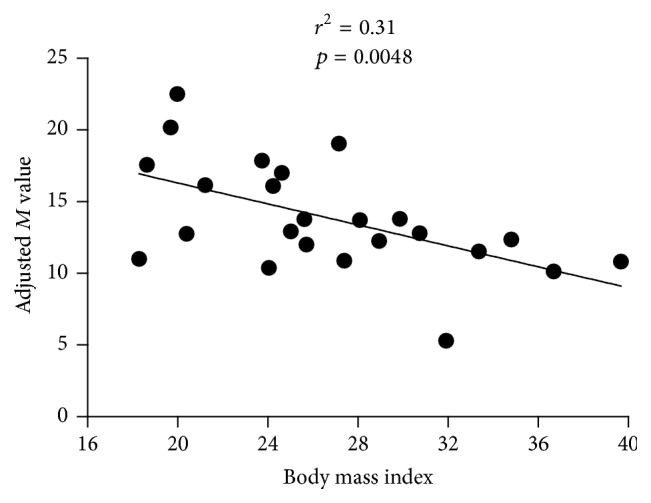
Graph showing the regression of the adjusted *M* value against BMI.

Figure 2 shows the regression between the lean body mass adjusted *M* value and the BMI of our study population (*r* ^2^ = 0.31, *p* = 0.0048).

## 4. Discussion

This study evaluated the variation in insulin sensitivity in women living with polycystic ovarian syndrome in relation to obesity. In our study, *M* value and *M* adjusted to lean mass were lower in both obese and nonobese people living with PCOS.

These findings are similar to those found in study populations of various ethnic groups using the euglycemic-hyperinsulinemic clamp technic. They found that in their populations women with PCOS were more insulin resistant even when matched for BMI with controls. Furthermore, they showed that body fat contributes to determining insulin resistance in these subjects [14, 15].

The fact that both obese and nonobese women living with PCOS were less insulin sensitive than their controls shows that although obesity may play a role in insulin resistance seen in this syndrome, there are other factors associated with PCOS implicated.

There is an increase in insulin levels in PCOS subjects. Insulin potentiates steroidogenic response to gonadotrophins both in vivo and in vitro; hence, during hyperinsulinemia there will be elevated androgen levels. This increased androgen activity is associated with insulin resistance [16, 17]. Insulin also increases the number of LH receptors in granulosa cells and in concert with FSH increases LH-binding capacity and is one reason for the elevated LH seen in PCOS. This elevated LH on its own increases androgen levels via its involvement in androstenedione production [17]. Insulin response to an oral glucose load is higher in lean and obese PCOS women compared to healthy ones [6]. This clearly signifies that, irrespective of the presence or absence of obesity, insulin resistance is associated with PCOS.

The lower insulin sensitivity in obese PCOS women compared to nonobese PCOS women shows that though obesity may not be the only cause of insulin resistance in PCOS subjects, it worsens the insulin resistance in PCOS [18] and therefore should be a treatment goal. Effectively, it has been shown that weight loss in obese PCOS subjects even without other therapeutic agents can restore insulin sensitivity [19].

Body composition analysis in this study revealed that PCOS was associated with android obesity as shown by a disproportionally high waist circumference for a given BMI. This type of fat distribution was similarly found in Caucasian women even in nonobese PCOS women [20]. Abdominal obesity or central obesity is known to play an important role in insulin resistance. Race also plays an important role in severity of adipose tissue distribution as Africans are more prone to subcutaneous adiposity than Caucasians [21, 22]. We can conclude that though android obesity may not be the sole determinant of insulin resistance in PCOS it is an important one.

Our results showed no significant difference in the resting energy expenditure (REE) of the three groups. Similar results were obtained in another study where there was no difference in REE between PCOS subjects and controls even after adjusting for age and BMI [23]. However, these results are in contrast to those from other studies which showed that PCOS is associated with decreased REE and postprandial thermogenesis as a result of insulin resistance [24, 25]. This was probably due to our small sample size in comparison to the large study population in those studies. In addition, our population had a higher BMI than in the above-mentioned studies and this may explain the tendency for REE to increase among the PCOS women in our study.

It is useful to report the trend though because it suggests that the etiology of obesity is not due to REE although more studies should be performed on a larger cohort before drawing firm conclusions. The lack of a difference between the groups suggests that poor lifestyle habits (such as diet and inactivity) may remain the main predisposing factor of the obesity in PCOS women. This means that dietary intervention and increased physical activity can significantly help to improve insulin sensitivity and obesity in women living with PCOS.

## 5. Conclusion

We studied body composition, clamp-derived insulin sensitivity, and resting energy expenditure in obese and nonobese PCOS sub-Saharan women. Insulin sensitivity was decreased in nonobese PCOS women and further decreased in the presence of obesity without a clear relation with resting energy expenditure.

## Figures and Tables

**Table 1:** Clinical and biological characteristics of the study population.

Characteristics	Status			*p* value
	Obese PCOS (*n* = 6)	Non-obese PCOS (*n* = 8)	Nonobese, non-PCOS (*n* = 10)	
Age (years)	[26 (23–30)]	[27 (24–29)]	[23 (23-24)]	0.070
Body mass index (kg/m^2^)	34.1 [31.9–36.7]	26.4 [24.5–28.5]	22.5 [19.7–24.6]	0.0003
Waist circumference (cm)	99 [93–104]	87 [78–93]	77 [69–83]	0.0023
Waist-to-hip ratio	0.82 [0.80–0.84]	0.80 [0.76–0.88]	0.77 [0.73–0.79]	0.084
Fat mass (kg)	41.2 [30.2–47]	23.3 [17.2–26.5]	17.1 [10.8–21]	0.0015
Lean mass (kg)	56.3 [51–57.7]	47.4 [43.3–50.8]	45.9 [41.4–50.4]	0.0378
Fat (%)	40.8 [37.7–45.3]	31.1 [25.3–36.7]	26.3 [20.6–30.2]	0.0012
Serum creatinine (mg/L)	8.1 [7.1–9.0]	8.9 [7.8–9.2]	8.8 [7.5–9.3]	0.617
Blood urea nitrogen (g/L)	0.36 [0.32–0.4]	0.38 [0.34–0.42]	0.38 [0.35–0.39]	0.711

Systolic blood pressure (mmHg)	121 [110–124]	104 [102–119]	113 [103–116]	0.155
Diastolic blood pressure (mmHg)	78 [73–82]	66 [65–81]	73 [68–84]	0.367

Fasting plasma glucose (mg/dL)	96 [95–105]	103 [91–113]	92 [82–97]	0.235

Results expressed as median [interquartile range].

**Table 2:** Lifestyle habits of our study population.

Variables	PCOS+/OB+	PCOS+/OB−	PCOS−/OB−
*N*	6 (%)	8 (%)	10 (%)

*Fruits consumption/week *			
<2 days	1 (16.7)	4 (50)	4 (40)
≥2 days	5 (83.3)	4 (50)	6 (60)
*Vegetable consumption/week *			
<2 days	4 (66.7)	5 (62.5)	6 (60)
≥2 days	2 (33.3)	3 (37.5)	4 (40)
*Sporting activity/week*			
<2 days	3 (50)	7 (87.5)	8 (80)
≥2 days	3 (50)	1 (12.5)	2 (20)

**Table 3:** Unadjusted and fat-free mass adjusted insulin sensitivity across subgroups.

*M* value (mg/kg/min)	Status			*p* value
	Obese PCOS (*n* = 6)	Non-obese PCOS (*n* = 8)	Non-obese, non-PCOS (*n* = 10)	
Unadjusted to lean body mass	6.6 [5.5–7.3]	9.1 [7.7–10]	11.9 [9.4–14.5]	0.002
Adjusted to lean body mass	11.2 [10.1–12.4]	12.9 [12.1–13.8]	16.6 [13.8–17.9]	0.012

**Table 4:** Correlation between clinical and biological characteristics and fat-free mass adjusted insulin sensitivity.

Characteristic	Pearson's correlation coefficient	*p* value
Age	−0.12	0.57
REE	−0.28	0.18
Systolic blood pressure	−0.27	0.21
Diastolic blood pressure	−0.25	0.24
Fasting plasma glucose	−0.26	0.22
Body mass index	−0.56	0.005
Blood urea nitrogen	0.04	0.84
Serum creatinine	−0.09	0.67

## References

[1] Norman R. J., Dewailly D., Legro R. S., Hickey T. E. (2007). Polycystic ovary syndrome. *The Lancet*.

[2] Dunaif A., Fauser B. C. J. M. (2013). Renaming PCOS—a two-state solution. *The Journal of Clinical Endocrinology & Metabolism*.

[3] Booth A., Magnuson A., Foster M. (2014). Detrimental and protective fat: body fat distribution and its relation to metabolic disease. *Hormone Molecular Biology and Clinical Investigation*.

[4] Horejsi R., Möller R., Rackl S. (2004). Android subcutaneous adipose tissue topography in lean and obese women suffering from PCOS: comparison with type 2 diabetic women. *American Journal of Physical Anthropology*.

[5] Godoy-Matos A. F., Vaisman F., Pedrosa A. P., Farias M. L. F., Mendonça L. M. C., Pinheiro M. F. M. C. (2009). Central-to-peripheral fat ratio, but not peripheral body fat, is related to insulin resistance and androgen markers in polycystic ovary syndrome. *Gynecological Endocrinology*.

[6] Dunaif A. (1997). Insulin resistance and the polycystic ovary syndrome: mechanism and implications for pathogenesis. *Endocrine Reviews*.

[7] Li M., Youngren J. F., Dunaif A. (2002). Decreased Insulin Receptor (IR) autophosphorylation in fibroblasts from patients with PCOS: effects of serine kinase inhibitors and IR activators. *The Journal of Clinical Endocrinology and Metabolism*.

[8] Qiu H.-Y., Chu Y.-L., Li M., Sun Y.-Y., Li H.-F. (2005). Tyrosine phosphorylation and protein expression of insulin receptor substrate-2 in the adipose tissue from patients with polycystic ovary syndrome. *Zhonghua Fu Chan Ke Za Zhi*.

[9] Rotterdam ESHRE/ASRM-Sponsored PCOS Consensus Workshop Group (2004). Revised 2003 consensus on diagnostic criteria and long-term health risks related to polycystic ovary syndrome (PCOS). *Human Reproduction*.

[10] Escobar-Morreale H. F., Carmina E., Dewailly D. (2012). Epidemiology, diagnosis and management of hirsutism: a consensus statement by the Androgen Excess and Polycystic Ovary Syndrome Society. *Human Reproduction Update*.

[11] Alberti K. G. M. M., Zimmet P. Z. (1998). Definition, diagnosis and classification of diabetes mellitus and its complications. Part 1: diagnosis and classification of diabetes mellitus. Provisional report of a WHO consultation. *Diabetic Medicine*.

[12] Mbanya A., Ngandeu A., Kamwa V. (2016). Metabolic features associated with positivity to ZnT8 autoantibody in sub-Saharan African young-onset diabetes patients. *Diabetes & Metabolism*.

[13] Sobngwi E., Kengne A.-P., Echouffo-Tcheugui J. B. (2014). Fasting insulin sensitivity indices are not better than routine clinical variables at predicting insulin sensitivity among Black Africans: a clamp study in sub-Saharan Africans. *BMC Endocrine Disorders*.

[14] Stepto N. K., Cassar S., Joham A. E. (2013). Women with polycystic ovary syndrome have intrinsic insulin resistance on euglycaemic-hyperinsulaemic clamp. *Human Reproduction*.

[15] Tosi F., Di Sarra D., Kaufman J.-M. (2015). Total body fat and central fat mass independently predict insulin resistance but not hyperandrogenemia in women with polycystic ovary syndrome. *The Journal of Clinical Endocrinology & Metabolism*.

[16] Baskind N. E., Balen A. H. (2016). Hypothalamic-pituitary, ovarian and adrenal contributions to polycystic ovary syndrome. *Best Practice & Research Clinical Obstetrics & Gynaecology*.

[17] Poretsky L., Cataldo N. A., Rosenwaks Z., Giudice L. C. (1999). The insulin-related ovarian regulatory system in health and disease. *Endocrine Reviews*.

[18] Barber T. M., McCarthy M. I., Wass J. A. H., Franks S. (2006). Obesity and polycystic ovary syndrome. *Clinical Endocrinology*.

[19] Barber T. M., Dimitriadis G. K., Andreou A., Franks S. (2016). Polycystic ovary syndrome: insight into pathogenesis and a common association with insulin resistance. *Clinical Medicine*.

[20] Kirchengast S., Huber J. (2001). Body composition characteristics and body fat distribution in lean women with polycystic ovary syndrome. *Human Reproduction*.

[21] Albu J. B., Murphy L., Frager D. H., Johnson J. A., Pi-Sunyer F. X. (1997). Visceral fat and race-dependent health risks in obese nondiabetic premenopausal women. *Diabetes*.

[22] De Lucia Rolfe E., Ong K. K., Sleigh A., Dunger D. B., Norris S. A. (2015). Abdominal fat depots associated with insulin resistance and metabolic syndrome risk factors in black African young adults. *BMC Public Health*.

[23] Churchill S. J., Wang E. T., Bhasin G. (2015). Basal metabolic rate in women with PCOS compared to eumenorrheic controls. *Clinical Endocrinology*.

[24] Robinson S., Chan S.-P., Spacey S., Anyaoku V., Johnston D. G., Franks S. (1992). Postprandial thermogenesis is reduced in polycystic ovary syndrome and is associated with increased insulin resistance. *Clinical Endocrinology*.

[25] Georgopoulos N. A., Saltamavros A. D., Vervita V. (2009). Basal metabolic rate is decreased in women with polycystic ovary syndrome and biochemical hyperandrogenemia and is associated with insulin resistance. *Fertility and Sterility*.

